# Evaluation of COVID-19 impact on DELAYing diagnostic-therapeutic pathways of lung cancer patients in Italy (COVID-DELAY study): fewer cases and higher stages from a real-world scenario*

**DOI:** 10.1016/j.esmoop.2022.100406

**Published:** 2022-02-03

**Authors:** L. Cantini, G. Mentrasti, G.L. Russo, D. Signorelli, G. Pasello, E. Rijavec, M. Russano, L. Antonuzzo, D. Rocco, R. Giusti, V. Adamo, C. Genova, A. Tuzi, A. Morabito, S. Gori, N. La Verde, R. Chiari, A. Cortellini, V. Cognigni, F. Pecci, A. Indini, A. De Toma, E. Zattarin, S. Oresti, E.G. Pizzutilo, S. Frega, E. Erbetta, A. Galletti, F. Citarella, S. Fancelli, E. Caliman, L. Della Gravara, U. Malapelle, M. Filetti, M. Piras, G. Toscano, L. Zullo, M. De Tursi, P. Di Marino, V. D’Emilio, M.S. Cona, A. Guida, A. Caglio, F. Salerno, G. Spinelli, C. Bennati, F. Morgillo, A. Russo, C. Dellepiane, I. Vallini, V. Sforza, A. Inno, F. Rastelli, V. Tassi, L. Nicolardi, V. Pensieri, R. Emili, E. Roca, A. Migliore, T. Galassi, M. L. Bruno Rocchi, R. Berardi

**Affiliations:** 1 Department of Medical Oncology, Università Politecnica delle Marche, AOU Ospedali Riuniti di Ancona, Ancona, Italy; 2 Oncologia Medica 1, Fondazione IRCCS Istituto Nazionale dei Tumori di Milano, Milan, Italy; 3 Niguarda Cancer Center, Grande Ospedale Metropolitano Niguarda, Milan, Italy; 4 Department of Surgery, Oncology and Gastroenterology, University of Padova, Padua, Italy; 5 Medical Oncology 2, Istituto Oncologico Veneto IRCCS, Padua, Italy; 6 Medical Oncology Unit, Fondazione IRCCS Ca' Granda Ospedale Maggiore Policlinico, Milan, Italy; 7 Department of Medical Oncology, Campus Bio-Medico University, Rome, Italy; 8 Medical Oncology Unit, Careggi University Hospital, Florence, Italy; 9 Department of Pulmonology and Oncology, AORN dei Colli Monaldi, Naples, Italy; 10 UOC Oncologia Medica, Azienda Ospedaliero Universitaria Sant'Andrea, Università La Sapienza, Rome, Italy; 11 Oncologia Medica, A.O.Papardo & Università di Messina, Messina, Italy; 12 UOC Clinica di Oncologia Medica, IRCCS Ospedale San Martino, Department of Internal Medicine and Medical Specialties (DIMI), Università degli Studi di Genova, Genoa, Italy; 13 Oncologia Medica, ASST Sette Laghi, Varese, Italy; 14 Thoracic Medical Oncology, Istituto Nazionale Tumori “Fondazione G Pascale”, IRCCS, Naples, Italy; 15 UOC Oncologia Medica, IRCCS Ospedale Sacro Cuore Don Calabria, Negrar di Valpolicella, Verona, Italy; 16 Department of Oncology, Ospedale Luigi Sacco, ASST Fatebenefratelli Sacco, Milan, Italy; 17 Medical Oncology, Ospedali Riuniti Padova Sud, Monselice, Italy; 18 Medical Oncology, St Salvatore Hospital, L’Aquila, Italy; 19 Department of Oncology and Hemato-Oncology, Università degli Studi di Milano, Milan, Italy; 20 Dipartment of Experimental Medicine, Università degli Studi della Campania “Luigi Vanvitelli”, Naples, Italy; 21 Department of Public Health, Università degli Studi di Napoli “Federico II”, Naples, Italy; 22 Oncologia Medica, A.O.Papardo, Messina, Italy; 23 UOC Oncologia Medica 2, IRCCS Ospedale San Martino, Genoa, Italy; 24 Department of Innovative Technologies in Medicine & Dentistry, Università G. D’Annunzio, Chieti-Pescara, Chieti, Italy; 25 UOC Pneumologia, Ospedale Mazzoni, Ascoli Piceno, Italy; 26 Oncologia Medica e Traslazionale, AO Santa Maria, Terni, Italy; 27 Department of Oncology, University of Turin, Ordine Mauriziano Hospital, Turin, Italy; 28 UOC Territorial Oncology, University “Sapienza”, AUSL Latina, Cds Aprilia, Aprilia, Italy; 29 Department of Onco-Hematology, AUSL della Romagna, Ravenna, Italy; 30 UOC Oncologia ed Ematologia, Department of Precision Medicine, Università degli studi della Campania “Luigi Vanvitelli”, Naples, Italy; 31 UOC Oncologia, Ospedale Mazzoni, Ascoli Piceno, Italy; 32 Chirurgia Toracica, AO Santa Maria, Terni, Italy; 33 Operative Oncology Unit, Azienda Ospedaliera Ospedali Riuniti Marche Nord, Pesaro, Italy; 34 Thoracic Oncology - Lung Unit, Pederzoli Hospital, Peschiera Del Garda, Italy; 35 Biomolecular Sciences Department, University of Urbino, Urbino, Italy

**Keywords:** lung cancer, COVID-19, diagnostic delay, therapeutic delay, staging

## Abstract

**Introduction:**

COVID-19 has disrupted the global health care system since March 2020. Lung cancer (LC) patients (pts) represent a vulnerable population highly affected by the pandemic. This multicenter Italian study aimed to evaluate whether the COVID-19 outbreak had an impact on access to cancer diagnosis and treatment of LC pts compared with pre-pandemic time.

**Methods:**

Consecutive newly diagnosed LC pts referred to 25 Italian Oncology Departments between March and December 2020 were included. Access rate and temporal intervals between date of symptoms onset and diagnostic and therapeutic services were compared with the same period in 2019. Differences between the 2 years were analyzed using the chi-square test for categorical variables and the Mann–Whitney *U* test for continuous variables.

**Results:**

A slight reduction (−6.9%) in newly diagnosed LC cases was observed in 2020 compared with 2019 (1523 versus 1637, *P* = 0.09). Newly diagnosed LC pts in 2020 were more likely to be diagnosed with stage IV disease (*P* < 0.01) and to be current smokers (someone who has smoked more than 100 cigarettes, including hand-rolled cigarettes, cigars, cigarillos, in their lifetime and has smoked in the last 28 days) (*P* < 0.01). The drop in terms of new diagnoses was greater in the lockdown period (percentage drop −12% versus −3.2%) compared with the other months included. More LC pts were referred to a low/medium volume hospital in 2020 compared with 2019 (*P* = 0.01). No differences emerged in terms of interval between symptoms onset and radiological diagnosis (*P* = 0.94), symptoms onset and cytohistological diagnosis (*P* = 0.92), symptoms onset and treatment start (*P* = 0.40), and treatment start and first radiological revaluation (*P* = 0.36).

**Conclusions:**

Our study pointed out a reduction of new diagnoses with a shift towards higher stage at diagnosis for LC pts in 2020. Despite this, the measures adopted by Italian Oncology Departments ensured the maintenance of the diagnostic-therapeutic pathways of LC pts.

## Highlights

• The COVID-19 outbreak had an impact on access to lung cancer (LC) diagnosis and treatment.
• A slight reduction (−6.9%) in newly diagnosed LC cases was observed in 2020 compared with 2019.
• Newly diagnosed LC pts in 2020 were more likely to be diagnosed with stage IV disease.
• The Italian Oncology Departments ensured the maintenance of the diagnostic-therapeutic pathways of LC pts.
• A reverse migration from high-volume to low-volume cancer centers was noted during the pandemic.

## Introduction

Since the beginning of 2020, COVID-19 has abruptly spread worldwide, becoming a global health emergency. Italy was among the most affected countries in terms of COVID-19-related new cases and deaths, especially during the first pandemic wave.^1^ Therefore, the Italian government introduced a national lockdown between 8 March and 4 May 2020, to minimize the human-to-human viral transmission and to limit as far as possible pandemic incidence and mortality.

The COVID-19 pandemic overwhelmed the whole health care system that has been forced to rapidly reorganize to this unprecedented scenario. Human and economic resources have been channeled to COVID-19 patient (pt) care pathways, while many diagnostic and therapeutic services have been deferred or cancelled in non-COVID-19-related care activities.^2^^,^^3^

In the setting of cancer pts care, many efforts have been placed in order to ensure high-quality standards for diagnostic-therapeutic pathways, according to the guidelines from the major scientific societies.^4^^,^^5^ Oncologic departments have experimented a substantial reorganization in management and maintenance of life-saving treatments, such as systemic therapies and radiotherapy.^6^, ^7^, ^8^ Recent studies have pointed out a remarkable reduction of new cancer diagnoses in Europe and USA during the pandemic period.^9^^,^^10^ Undiagnosed cancer diseases are expected to emerge at a more advanced stage and with a worst prognosis,^11^ including that a significant delay in diagnosis and access to treatment may result in suboptimal therapeutic care of cancer pts and then in increased mortality.^12^^,^^13^

Lung cancer (LC) represents the leading cause of cancer-related deaths worldwide and usually is diagnosed at an advanced stage.^14^ Considering the clinical spectrum and potential overlap of LC symptoms and radiological findings with COVID-19 disease, the differential diagnosis may be complicated and challenging.^15^

In this study, using datasets from different Italian oncologic departments, we aimed to assess whether the COVID-19 outbreak had an impact on new diagnoses of LC. For this purpose, we evaluated access to diagnosis and treatment of a cohort of newly diagnosed LC pts during the pandemic and compared it with the pre-pandemic period, to provide a real-world picture of efficacy of the health care system response to the COVID-19.

## Material and methods

### Study design and population

The COVID-DELAY (‘Evaluation of COVID-19 impact on DELAYing diagnostic-therapeutic pathways of lung cancer patients in Italy’) study was a multicentric, observational, retrospective study. The primary objective of the study was to assess whether the COVID-19 outbreak had an impact on LC pts’ likelihood of receiving timely diagnosis and access to treatment in 2020, by assessing the total number of new diagnoses, access rate (number of pts/month), and temporal intervals between date of symptoms onset, diagnosis, first oncological appointment, treatment start, and first radiological reassessment and comparing with those of the same period in 2019. The secondary objective was to evaluate whether the COVID outbreak had an impact on the stage of disease of new diagnoses of LC in 2020 compared with 2019.

**Figure 1:**
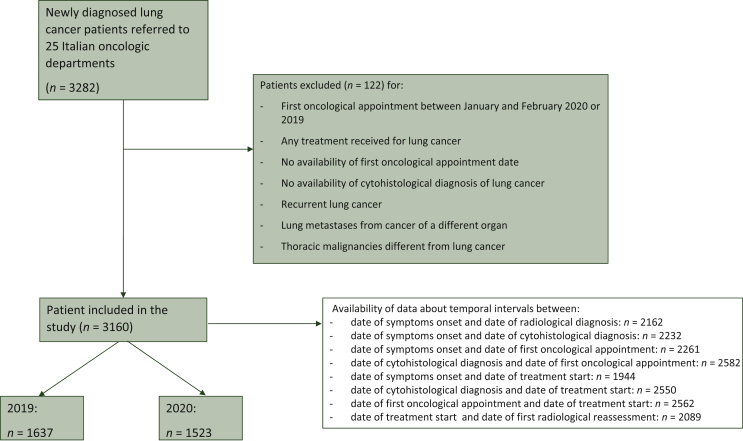
**STROBE diagram.** Identification and selection of study population according to inclusion and exclusion criteria.

Clinical records of all consecutive newly diagnosed LC pts referred to 25 Italian Oncology Departments (Supplementary Table S1, available at https://doi.org/10.1016/j.esmoop.2022.100406) between March and December 2020 and between March and December 2019 were reviewed. The inclusion criteria were: (i) pts, aged 18 years or older, with histologically or cytologically proven diagnosis of LC (either non-small-cell LC, NSCLC or small-cell LC, SCLC) between March and December 2020 or March and December 2019, (ii) at least one type of oncological treatment (surgery, radiotherapy, or systemic therapy) received after diagnosis, (iii) availability of data about radiological diagnosis, cytohistological diagnosis, and treatment start. Patient data were not collected if they had recurrent LC, lung metastases from cancer of a different organ, or different thoracic malignancies (lymphoma, thymic cancer, and malignant pleural mesothelioma). Mean monthly access rate (number of pts/month) and temporal intervals between date of symptoms onset, radiological diagnosis, cytohistological diagnosis, first oncological appointment, treatment start, and first radiological reassessment in 2020 were computed and compared with those of the same period in 2019. Data of pts who had their LC diagnosis after the first oncological appointment (as per standard practice of referral hospitals) were not included in the calculation of these specific temporal intervals to avoid negative values (Figure 1). Baseline (at diagnosis) data about age, sex, province, smoking status (including all tobacco), Eastern Cooperative Oncology Group performance status (ECOG PS), tumor histological and molecular subtype [including mutational status and programmed death-ligand 1 (PD-L1) positivity], and tumor stage were collected. Treatment setting (neoadjuvant, adjuvant, metastatic) was also retrieved from medical records and differences between the 2 years were analyzed.

Subgroup analyses were also carried out by specifically looking at the lockdown period and at the infection rate of the provinces where LC pts were diagnosed (high- versus medium/low-infected provinces)^16^ to assess whether restrictive measures initially adopted by the Italian Government had a major impact on the total number of diagnoses. The reference time period for the lockdown was established as 1 April to 30 June 2020 (instead of 8 March to 4 May 2020), since a conventional time interval of about 1 month between diagnosis and first oncological appointment was expected. Pts were then categorized according to the number of new LC diagnoses in the hospital where they were treated: high volume (≥150 new diagnoses in the investigated 2-year period) versus low/medium volume (<150 diagnoses).

Ethical approval to conduct this study was obtained by the respective local ethical committees on human experimentation of each participating center, after previous approval by the coordinating center (‘Comitato Etico Regionale delle Marche - C.E.R.M.’, Reference Number 2021 139). The authors are accountable for all aspects of the work in ensuring that questions related to the accuracy or integrity of any part of the work are appropriately investigated and resolved. The study was conducted in accordance with the precepts of Good Clinical Practice and the ethical principles of the Declaration of Helsinki. Written informed consent was provided by all patients. Results presented in this article contain no personally identifiable information from the study.

### Sample size estimation

To estimate the study sample size, a 20% reduction of newly diagnosed LC cases in the pandemic year (2020) compared with 2019 was postulated. Therefore, assuming a 95% confidence interval range of 10% (±5%), data of at least 250 newly diagnosed LC pts were required in 2019, corresponding to 200 new diagnoses in 2020.

### Statistical analysis

Baseline patient and disease characteristics, together with treatment information, were reported using descriptive statistics. Categorical variables were reported as either fractions or percentage; continuous variables either as mean, standard deviation (if normally distributed), or as median and interquartile range (if not-normally distributed). Differences between 2020 and 2019 were analyzed using the Fisher’s exact test or chi-square test for categorical variables and the paired Student’s *t*-test or Mann–Whitney *U* test for continuous variables as appropriate. All statistical analyses were carried out using R 3.6.0 (R Foundation for Statistical Computing, Vienna, AT) and a two-sided *P* value of <0.05 was deemed statistically significant. The data-wrapper software (https://www.datawrapper.de) was used to create the graph maps.

## Results

### Population

**Figure 2:**
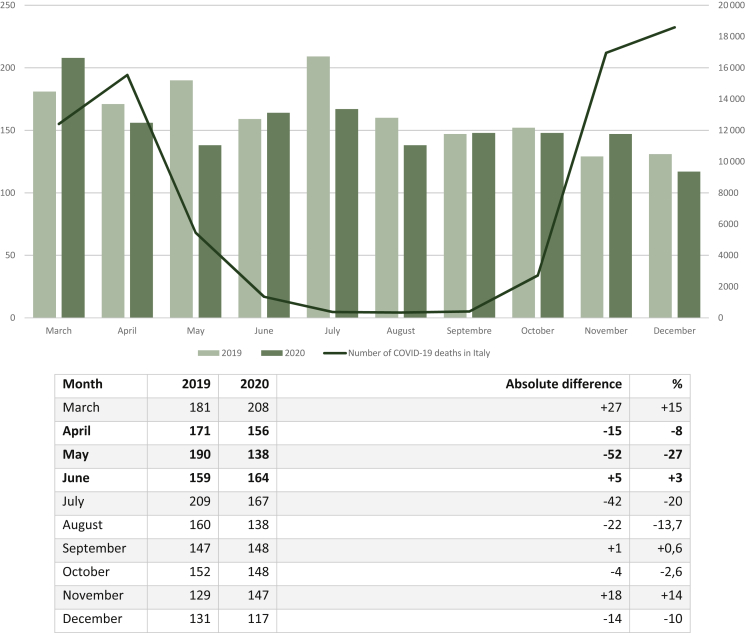
**Monthly differences of new lung cancer diagnoses between 2019 and 2020.** April, May, and June 2020 (in bold type) were considered as the lockdown timeframe.

A total of 3160 pts met our inclusion criteria (Figure 1). A slight, albeit non-significant reduction (−6.9%) in newly diagnosed LC cases was seen in 2020 (*n* = 1523) when compared with 2019 (*n* = 1637). The mean monthly access rates were 163.7 versus 152.3, respectively (access rate ratio = 0.93, *P* = 0.09) (Figure 2). Median age was similar (69 years old, *P* = 0.96) between the two cohorts. The percentage of female pts (36% versus 37%) was also similar between the two cohorts (*P* = 0.53). Most NSCLC pts had adenocarcinoma as tumor histology, and the percentage was higher in 2020 (65% versus 61%, *P* = 0.04). Additionally, smoking history differed in 2020, with more LC pts currently smoking at time of diagnosis (39% versus 34%, *P* < 0.01).

Most strikingly, clinical stage at diagnosis differed between 2020 and 2019. Specifically, after the COVID-19 outbreak, pts were more likely to be diagnosed with stage IV disease (72% versus 67%, *P* < 0.01). Early-stage LC was represented in a lower proportion of newly diagnosed pts in the 2020 versus 2019 cohort. The ECOG PS at the start of treatment was similar between the two cohorts (*P* = 0.17), yet a higher percentage of LC pts presented with poor condition (ECOG PS > 2, 3.5% versus 2%, *P* = 0.04). The more advanced stage did not seem to affect the treatment setting which was similar between cohorts (*P* = 0.50 for NSCLC, *P* = 0.72 for SCLC), nor the molecular profile with a similar number of pts with tumors harboring targetable alterations (19% versus 19%) and PD-L1 positivity (64% versus 61%, *P* = 0.15).

Similarly, the pandemic did not have an impact on the multidisciplinary management of LC pts, with 44% of the cases being discussed in multidisciplinary meetings in 2020 compared with 45% in 2019. A slight reduction of LC pts treated in the context of clinical trials was seen, however, during 2020 (5% versus 7%, *P* = 0.07). Demographic, clinicopathological, and treatment characteristics by year of treatment are summarized in Table 1.

**Table 1:** Patient baseline characteristics by year of diagnosis

Characteristic	2019	2020	*P* value^a^
Patients, *n*	1637	1523	
Monthly access rate, *n*	163.7	152.3	0.09
Median age, years (IQR)	69 (13)	69 (14)	0.96
Male, *n* (%)	1028 (63)	974 (64)	0.53
Smoking status, *n* (%)			<0.01^b^
Current smoker	550 (34)	589 (39)	
Former smoker	834 (52)	697 (46)	
Never smoker	212 (14)	220 (15)	
Asymptomatic disease onset, *n* (%)	243 (19)	300 (21)	<0.01^b^
Tumor histology			0.04^b^
NSCLC adenocarcinoma, *n* (%)	1007 (61)	984 (65)	
NSCLC squamocellular carcinoma, *n* (%)	303 (19)	267 (18)	
NSCLC others, *n* (%)	142 (9)	90 (6)	
SCLC, *n* (%)	180 (11)	175 (11)	
Stage at diagnosis, *n* (%)			<0.01^b^
Stage I	106 (6)	71 (5)	
Stage II	126 (8)	69 (4)	
Stage III	304 (19)	281 (19)	
Stage IV	1083 (67)	1076 (72)	
Targetable driver mutations (EGFR, ALK, ROS1), *n* (%)	212 (19)	189 (19)	0.76
PD-L1 status, *n* (%)			0.23
Negative	456 (39)	401 (36)	
1%-49%	350 (30)	366 (33)	
≥50%	364 (31)	346 (31)	
Treatment setting (NSCLC), *n* (%)			0.50
Adjuvant	137 (10)	121 (9)	
Neoadjuvant	59 (5)	44 (5)	
Locally advanced	137 (10)	120 (9)	
Metastatic	1001 (75)	969 (77)	
Treatment setting (SCLC), *n* (%)			0.72
Limited disease	28 (18)	30 (20)	
Extensive disease	124 (82)	115 (80)	
MTD discussion	738 (45)	663 (44)	0.36
Treatment within clinical trials	116 (7)	84 (5)	0.07
ECOG PS at start of treatment, *n* (%)			0.17
0	504 (33)	455 (31)	
1	743 (49)	724 (49)	
2	241 (15)	239 (16)	
3	28 (2)	46 (3)	
4	3 (1)	2 (1)	

Current smoker, someone who has smoked more than 100 cigarettes, including hand-rolled cigarettes, cigars, cigarillos, in their lifetime and has smoked in the last 28 days; Former smoker, someone who has smoked more than 100 cigarettes in their lifetime but has not smoked in the last 28 days; Never smoker, someone who has not smoked more than 100 cigarettes in their lifetime and does not currently smoke; ALK, anaplastic lymphoma kinase; ECOG PS, Eastern Cooperative Oncology Group performance status; EGFR, epidermal growth factor receptor; IQR, interquartile range; MDT, multidisciplinary team; NSCLC, non-small-cell lung cancer; PD-L1, programmed death-ligand 1; ROS1, proto-oncogene tyrosine-protein kinase ROS; SCLC, small-cell-lung cancer.

a Chi-square test comparing proportions between 2019 and 2020. *P* values were calculated excluding unknown values.

b Statistically significant (*P* < 0.05).

### Time intervals

Looking at access to cancer diagnosis, staging, and treatment of LC pts after March 2020, no major differences emerged compared with pre-pandemic time (Table 2 and Supplementary Figure S1, available at https://doi.org/10.1016/j.esmoop.2022.100406). In particular, time intervals between symptoms onset and radiological diagnosis (median 28 versus 28 days, *P* = 0.94), symptoms onset and cytohistological diagnosis (49 versus 48 days, *P* = 0.92) were similar between the two years. Similarly, no difference was present in the time interval between symptom onset and first oncological appointment (63 versus 65 days, *P* = 0.06). The interval between cytohistological diagnosis and first oncological appointment was even shorter in 2020 (20 versus 24 days, *P* < 0.01).

**Table 2:** Temporal intervals between date of symptoms onset, radiological diagnosis, cytohistological diagnosis, first oncological appointment, treatment start, and first radiological reassessment by year of diagnosis

Time interval	2019 Median, days (IQR)	2020 Median, days (IQR)	*P* value^a^
Symptom onset/radiological diagnosis	28 (46)	28 (49)	0.94
Symptom onset/cytohistological diagnosis	48 (55)	49 (57)	0.92
Symptom onset/first oncological appointment	65 (72)	63 (71.2)	0.06
Cytohistological diagnosis/first oncological appointment	24 (25)	20 (22)	<0.01^b^
Symptom onset/treatment start	83 (71.2)	78.5 (72)	0.40
Cytohistological diagnosis/treatment start	35 (32)	31 (29)	<0.01^b^
First oncological appointment/treatment start	16 (21)	15 (20)	0.45
Treatment start/first radiological evaluation	71 (32.2)	71.5 (36)	0.33

IQR, interquartile range.

a Mann-Whitney U test comparing time intervals between 2019 and 2020. *P* values were calculated excluding patients with unknown values. Data of patients who had their lung cancer diagnosis after first oncological appointment (as per standard practice of referral Hospitals) were also excluded in the calculation of these specific temporal intervals.

b Statistically significant (*P* < 0.05).

Looking more specifically at therapeutic pathways of LC pts, the emergency response adopted during the pandemic resulted in LC pts receiving timely treatment and follow-up. In particular, 2020 and 2019 time intervals between symptoms onset and treatment start (median 78.5 versus 83 days, *P* = 0.40), first oncological appointment and treatment start (15 versus 16 days, *P* = 0.45), treatment start and first radiological revaluation (71 versus 71 days, *P* = 0.36) were similar, with the time interval between cytohistological diagnosis and treatment start even shorter (31 versus 35 days, *P* < 0.01).

### Subgroup analysis

Given that the impact of the COVID-19 pandemic on LC diagnoses might have differed according to time of the year, provincial infection rate, and hospital volume, sensitivity analyses were conducted in some subgroups of pts.

**Figure 3:**
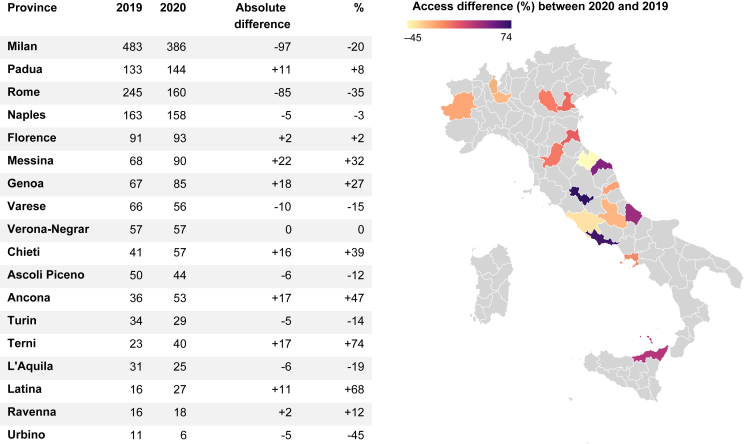
**Differences of new lung cancer diagnoses between 2019 and 2020 by provinces included in our Italian multicentric study**.

The drop in terms of new diagnoses was greater in the lockdown period compared with the other months that were analyzed, albeit the difference was not significant (mean of monthly differences: −22.6 diagnoses versus −6.57, percentage drop −12% versus −3.2%, *P* = 0.22). May 2020 was the month with the greatest drop of new LC diagnoses (−27%) (Figure 2). Looking at the percentage of pts referred to hospitals in high-infected versus low/medium-infected provinces, no significant difference was noted during the pandemic compared with 2020 (44% versus 46%, *P* = 0.24). Rome (−85, −35%) and Milan (−97, −20%) were the two provinces with the most prominent drop in terms of absolute values (Figure 3). In addition, more LC pts were referred to low/medium volume hospitals in 2020 compared with 2019 (36% versus 31%, *P* = 0.01).

## Discussion

By March 2020, the COVID-19 outbreak would have unprecedentedly changed the face of cancer care and permanently shaped the global health care scenery. With Italy being one of the most heavily affected countries, Italian medical oncologists were expected to lead their pts through the eye of the storm.^7^

Right alongside us, cancer pts have unexpectedly ended up fighting a twofold battle: on one hand facing the fear of contracting COVID-19 in order to keep their treatment going, and on the other hand dealing with the uncertainty of deferred elective oncological procedures as well as treatment plan adaptations. In managing such vulnerable populations, ‘the sooner, the better’ principle (in terms of disease stage at diagnosis and timely access to treatment) stands firm despite the gap in disaster preparedness.^17^^,^^18^

Since the first pandemic wave with a health care system close to collapse and limited experience-based guidelines to keep the cancer care ship afloat, medical oncologists’ associations had to elaborate a prompt response. Conflicting measures have been adopted to effectively exit the crisis, such as patient-tailored reconsideration of treatment schedules to reduce avoidable hospital admissions, visits’ conversion to telehealth encounters, and multidisciplinary board rearrangements following specialists’ reallocation to COVID-19 units.

The scientific community now wonders which effects on the expected cancer incidence and mortality rates we are going to reap in the near future.^11^^,^^19^ Despite the earliest establishment of experts’ consensus and the implementation of these recommendations in daily clinical practice, the outcome of the efforts made to prevent diagnostic delays and the much-feared ‘upstaging effect’ are still a matter of speculation and might affect the years to come.^4^^,^^6^^,^^20^ Although a growing number of publications has focused on continuum of care impairment during the first peaks of coronavirus spread,^21^^,^^22^ there is still a great deal of uncertainty on how COVID-19 has impacted cancer diagnosis, staging, and time to treatment initiation after March 2020.

To our knowledge, this is the first report to provide a thorough insight on whether the measures adopted by the Italian oncology departments in response to the COVID-19 outbreak were able to optimally address quality of care issues and impact LC pts’ likelihood of receiving timely diagnosis and treatment compared with pre-pandemic time.

According to previous findings that revealed an upsetting drop in the number of new incidence cancers globally,^9^^,^^10^^,^^23^ our study confirmed a reduction (−6.9%) in LC diagnoses in 2020 (*n* = 1523) compared with 2019 (*n* = 1637) in Italy. With the pandemic’s challenges considered, this decline in the rate of newly diagnosed LC is consistent with the results by London et al.^24^ Similarly, their 2020-2019 network comparison of 20 United States health care institutions documented that the decrease in new incidence neoplasms varied considerably among different cancer types, with LC as the least affected, possibly due to the absence of validated screening programs.

A feasible explanation could be also traced to the majority of current smokers at time of diagnosis in 2020 (39% versus 34%, *P* < 0.01). Considering the clinical spectrum of LC onset, especially when smoke-related, and its potential overlap with COVID-19 disease, the convergence between the virus and the smoke-induced tumor’s manifestations may have contributed to mitigate the pandemic’s effects on LC diagnosis compared with other malignancies. As shown in the TERAVOLT study by Garassino et al.*,*^25^ the majority of pts with thoracic cancers and COVID-19 were current smokers (someone who has smoked more than 100 cigarettes, including hand-rolled cigarettes, cigars, cigarillos, in their lifetime and has smoked in the last 28 days) or former smokers (someone who has smoked more than 100 cigarettes in their lifetime but has not smoked in the last 28 days), but only smoking history in the multivariate analysis was associated with increased risk of death.

Strikingly relevant from our data is the significant difference in the clinical stage at diagnosis between the 2 years. Unprecedentedly, our results demonstrate that LC pts were more likely detected at stage IV in 2020 compared with pre-pandemic time (72% versus 67%, *P* < 0.01). Although the consequent impact of such post-pandemic later stage diagnosis on survival is still to be determined, some concerning predictions from a large UK modeling study have estimated an increase in LC mortality rate of ∼5% up to 5 years after diagnosis.^12^^,^^26^

Our analysis shows that, despite great difficulties, no flaw in the multidisciplinary management system has been exposed during the pandemic (44% of cases discussed in 2020 tumor boards versus 45% in 2019). Specifically, looking at temporal intervals in the diagnostic-therapeutic pathway (date of symptoms onset, radiological diagnosis, cytohistological diagnosis, treatment start and first radiological revaluation), no gaps at any level emerged from our data. The absence of a difference between symptom onset and the first radiological examination, despite higher stages at diagnosis, could have been partially influenced by the interindividual variability in reporting symptoms onset. According to our findings, the Italian COVINT study also observed a small rate (8.9%) of deferred anticancer treatment because of the pandemic.^27^

Our study has revealed a setback in LC pts’ participation in clinical trials after COVID-19 (5% versus 7%, *P* = 0.07). Whereas it is too soon to tell how the slowdowns within the cancer research community will affect the progress in cancer care, an extensive analysis supported by the National Institutes of Health (NIH) confirmed that coronavirus has globally hampered the enrollment in clinical studies, with a drop of trials completion rate between 13% and 23% from April to October 2020.^28^

Since the present study represents the joint effort of a nationwide cooperation, it also accounts for regional variations in the response to the pandemic. Therefore, it should not represent differences in cancer incidence throughout the country. Of note, we observed a reverse migration from high-volume cancer centers to low-volume oncology departments in 2020 compared with the previous year, irrespective of the spatial heterogeneity of the infection spread. As an adjustment to a pandemic context, this proved decentralization of cancer care might represent the epiphenomenon of lockdowns institution and confinement measures after COVID-19.

We acknowledge that our work has potential limitations as a retrospective study. As our analysis did not include all centers and LC patients in Italy, the observed reduction in newly diagnosed LC might, for example, merely reflect an additional shift of patients towards (low) volume centers not participating in the study. Gathering broad national collaboration and extensive case series, however, we consider these results as an accurate mirror of our country’s reality after March 2020. Moreover, as stated by Sharpless^11^ and Curigliano et al.^6^ different oncological settings could have been variably affected during the COVID-19 pandemic. Including advanced, neoadjuvant, and adjuvant disease, all characterized by very distinct clinical presentation and outcomes, our study was expected to overcome this limit.

From our results we can conclude that, while COVID-19 repercussions on cancer care will likely be felt for decades to come, Italian medical oncologists set a virtuous example to address quality of care issues and ensure timely diagnosis and treatment of LC pts after March 2020. As the pandemic shows no sign of abating, the strategy developed to answer the emergency may prove even more valuable to take further steps towards maintaining high-quality standards for diagnostic-therapeutic pathways. More importantly, our findings stress the value of keeping the performance bar high for cancer patients in order to avoid the dire consequences of a cancer pandemic once the COVID-19 pandemic would be over. Future investigations will offer a more exhaustive and long-term picture on the effectiveness of the efforts made to also contain the coronavirus tidal wave in other cancer settings.
