# Erratum to ‘Evaluation of COVID-19 impact on DELAYing diagnostic-therapeutic pathways of lung cancer patients in Italy (COVID-DELAY study): fewer cases and higher stages from a real-world scenario’ [ESMO Open Volume 7, Issue 2, April 2022, 100406]

**DOI:** 10.1016/j.esmoop.2022.100471

**Published:** 2022-04-01

**Authors:** L. Cantini, G. Mentrasti, G. Lo Russo, D. Signorelli, G. Pasello, E. Rijavec, M. Russano, L. Antonuzzo, D. Rocco, R. Giusti, V. Adamo, C. Genova, A. Tuzi, A. Morabito, S. Gori, N. La Verde, R. Chiari, A. Cortellini, V. Cognigni, F. Pecci, A. Indini, A. De Toma, E. Zattarin, S. Oresti, E.G. Pizzutilo, S. Frega, E. Erbetta, A. Galletti, F. Citarella, S. Fancelli, E. Caliman, L. Della Gravara, U. Malapelle, M. Filetti, M. Piras, G. Toscano, L. Zullo, M. De Tursi, P. Di Marino, V. D’Emilio, M.S. Cona, A. Guida, A. Caglio, F. Salerno, G.P. Spinelli, C. Bennati, F. Morgillo, A. Russo, C. Dellepiane, I. Vallini, V. Sforza, A. Inno, F. Rastelli, V. Tassi, L. Nicolardi, M.V. Pensieri, R. Emili, E. Roca, A. Migliore, T. Galassi, M.B.L. Rocchi, R. Berardi

**Affiliations:** 1 Department of Medical Oncology, Università Politecnica delle Marche, AOU Ospedali Riuniti di Ancona, Ancona, Italy; 2 Oncologia Medica 1, Fondazione IRCCS Istituto Nazionale dei Tumori di Milano, Milan, Italy; 3 Niguarda Cancer Center, Grande Ospedale Metropolitano Niguarda, Milan, Italy; 4 Department of Surgery, Oncology and Gastroenterology, University of Padova, Padua, Italy; 5 Medical Oncology 2, Istituto Oncologico Veneto IRCCS, Padua, Italy; 6 Medical Oncology Unit, Fondazione IRCCS Ca' Granda Ospedale Maggiore Policlinico, Milan, Italy; 7 Department of Medical Oncology, Campus Bio-Medico University, Rome, Italy; 8 Medical Oncology Unit, Careggi University Hospital, Florence, Italy; 9 Department of Pulmonology and Oncology, AORN dei Colli Monaldi, Naples, Italy; 10 UOC Oncologia Medica, Azienda Ospedaliero Universitaria Sant'Andrea, Università La Sapienza, Rome, Italy; 11 Oncologia Medica, A.O.Papardo & Università di Messina, Messina, Italy; 12 UOC Clinica di Oncologia Medica, IRCCS Ospedale San Martino, Genoa, Italy; 13 Oncologia Medica, ASST Sette Laghi, Varese, Italy; 14 Thoracic Medical Oncology, Istituto Nazionale Tumori “Fondazione G Pascale”, IRCCS, Naples, Italy; 15 UOC Oncologia Medica, IRCCS Ospedale Sacro Cuore Don Calabria, Negrar di Valpolicella, Verona, Italy; 16 Department of Oncology, Ospedale Luigi Sacco, ASST Fatebenefratelli Sacco, Milan, Italy; 17 Medical Oncology, Ospedali Riuniti Padova Sud, Monselice, Italy; 18 Medical Oncology, St Salvatore Hospital, L’Aquila, Italy; 19 Department of Oncology and Hemato-Oncology, Università degli Studi di Milano, Milan, Italy; 20 Dipartment of Experimental Medicine, Università degli Studi della Campania “Luigi Vanvitelli”, Naples, Italy; 21 Department of Public Health, Università degli Studi di Napoli “Federico II”, Naples, Italy; 22 Oncologia Medica, A.O.Papardo, Messina, Italy; 23 UOC Oncologia Medica 2, IRCCS Ospedale San Martino, Genoa, Italy; 24 Department of Innovative Technologies in Medicine & Dentistry, Università G. D’Annunzio, Chieti-Pescara, Chieti, Italy; 25 UOC Pneumologia, Ospedale Mazzoni, Ascoli Piceno, Italy; 26 Oncologia Medica e Traslazionale, AO Santa Maria, Terni, Italy; 27 Department of Oncology, University of Turin, Ordine Mauriziano Hospital, Turin, Italy; 28 UOC Territorial Oncology, University “Sapienza”, AUSL Latina, Cds Aprilia, Aprilia, Italy; 29 Department of Onco-Hematology, AUSL della Romagna, Ravenna, Italy; 30 UOC Oncologia ed Ematologia, Department of Precision Medicine, Università degli studi della Campania “Luigi Vanvitelli”, Naples, Italy; 31 UOC Oncologia, Ospedale Mazzoni, Ascoli Piceno, Italy; 32 Chirurgia Toracica, AO Santa Maria, Terni, Italy; 33 Operative Oncology Unit, Ospedale Santa Maria della Misericordia, Urbino, Italy; 34 Thoracic Oncology - Lung Unit, Pederzoli Hospital, Peschiera Del Garda, Italy; 35 Biomolecular Sciences Department, University of Urbino, Urbino, Italy; 36 Department of Internal Medicine and Medical Specialties (DIMI), Università degli Studi di Genova, Genoa, Italy

The publisher regrets that at the time the article was published the name of the author N. La Verde was mistakenly abbreviated as N.L. Verde. This has now been corrected.

The publisher would like to apologise for any inconvenience caused.

