# Supplementary material for: Evaluation of COVID-19 impact on DELAYing diagnostic-therapeutic pathways of lung cancer patients in Italy (COVID-DELAY study): fewer cases and higher stages from a real-world scenario
Source: ESMO Open. 2022 Feb 3;7(2):100406. doi: 10.1016/j.esmoop.2022.100406 (PMC8810307; doi:10.1016/j.esmoop.2022.100406)
Supplement: Supplementary Table S1 and Figure S1 [file mmc1.docx]

| **Institution** | **Department** |
| --- | --- |
| Università Politecnica delle Marche, Ospedali Riuniti di Ancona, Ancona | Oncology Clinic |
| Ospedali Riuniti Padova Sud "Madre Teresa Di Calcutta", Monselice, Padova | Medical Oncology |
| San Salvatore Hospital, University of L'Aquila, L'Aquila | Medical Oncology |
| Università Degli Studi Della Campania "Luigi Vanvitelli", Napoli | Medical Oncology |
| "Luigi Sacco" Hospital-ASST Fatebenefratelli Sacco, Milano | Department of Oncology |
| University of Turin and AO Ordine Mauriziano, Torino | Medical Oncology |
| IRCCS Ospedale Sacro Cuore Don Calabria, Negrar di Valpolicella, Verona | Oncology Unit |
| SS Annunziata Hospital, Universitá G. D'Annunzio, Chieti | Medical Oncology |
| Ospedale Santa Maria delle Croci di Ravenna, Ravenna | Medical Oncology |
| IRCCS Istituto Nazionale dei Tumori, Fondazione "Pascale", Napoli | Medical Oncology |
| Azienda Ospedaliera Santa Maria di Terni, Terni | Department of Oncology |
| Aprilia Hospital, Aprilia | UOC Territorial Oncology of Aprilia |
| Ospedali dei Colli "Monaldi", Napoli | Pneumo-Oncology Unit |
| Ospedale Santa Maria della Misericordia di Urbino, Urbino | Medical Oncology |
| Istituto Oncologico Veneto IRCCS, Padova | Medical Oncology |
| Sant'Andrea Hospital of Rome, Roma | Medical Oncology Unit |
| Fondazione IRCCS Ca' Granda Ospedale Maggiore Policlinico, Milano | Medical Oncology Unit |
| IRCCS Ospedale Policlinico San Martino, Genova | Lung Cancer Unit |
| Fondazione IRCCS Istituto Nazionale dei Tumori, Milano | Department of Medical Oncology |
| ASST dei Sette Laghi, Varese | Medical Oncology |
| Campus Bio-Medico University, Roma | Medical Oncology |
| Azienda Ospedaliera Papardo and Università degli Studi di Messina, Messina | Medical Oncology |
| Careggi University Hospital, Firenze | Department of Medical Oncology |
| Grande Ospedale Metropolitano Niguarda, Milano | Niguarda Cancer Center |
| Ospedale Mazzoni, Ascoli Piceno | Medical Oncology |

**Supplementary Table 1. Departments participating in the COVID-DELAY study.**

**Supplementary Figure 1. Boxplots showing temporal intervals between date of symptoms onset, radiological diagnosis, cytohistological diagnosis, first oncological appointment, treatment start, and first radiological reassessment in 2019 and 2020.**

**
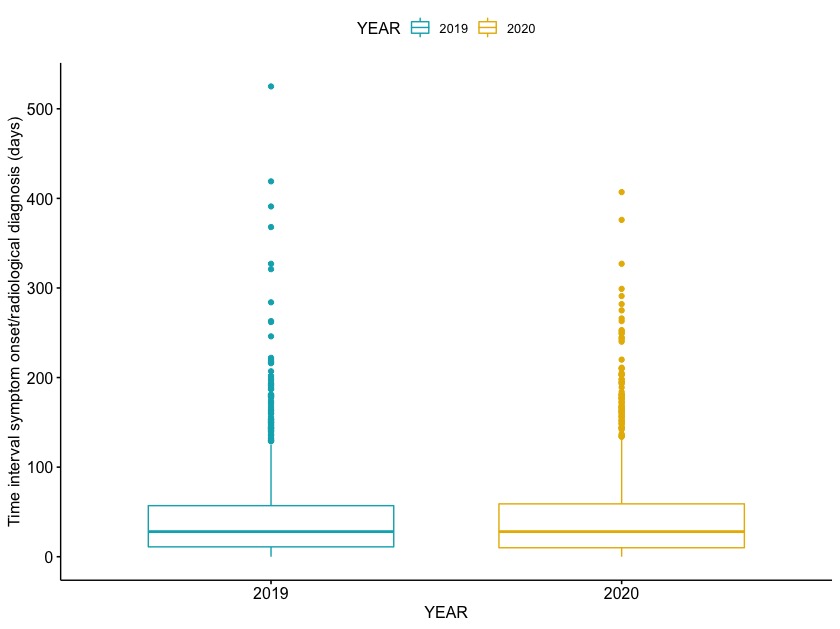

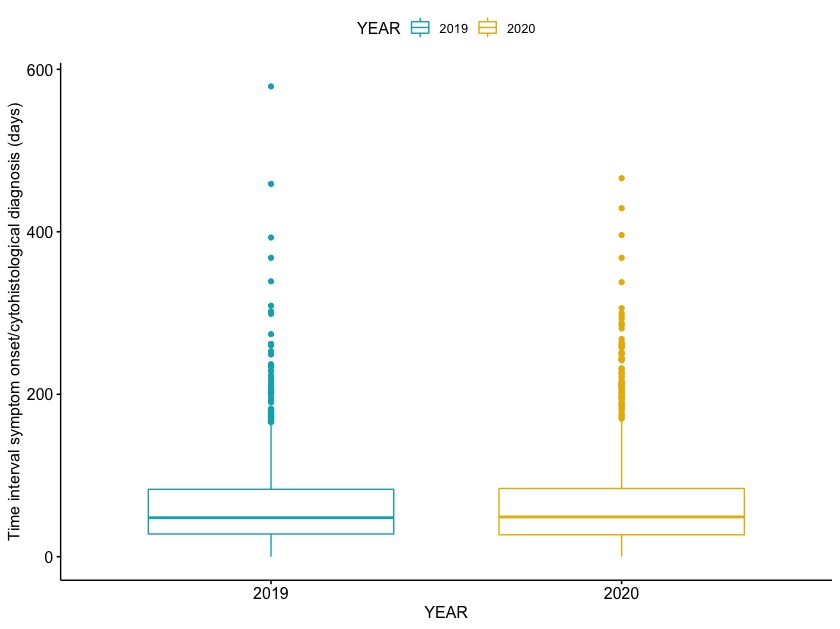

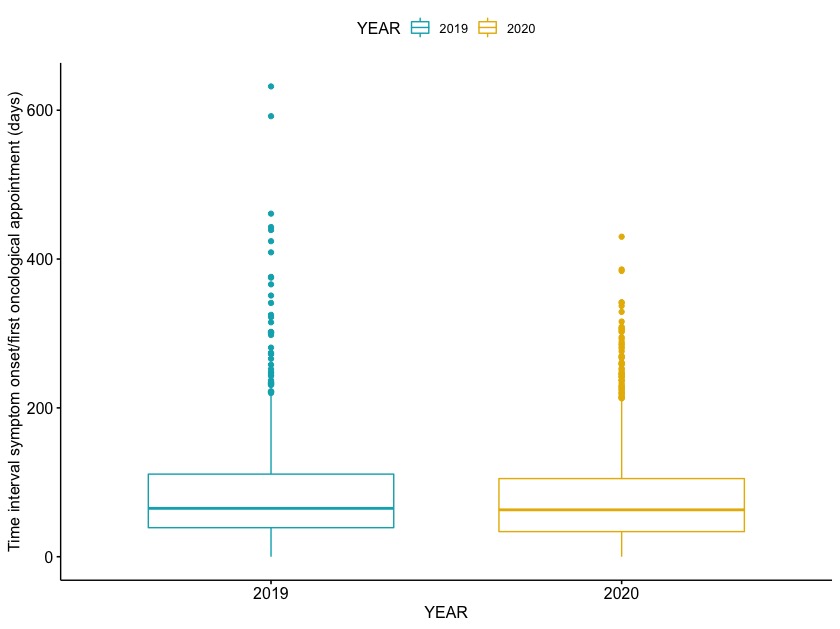

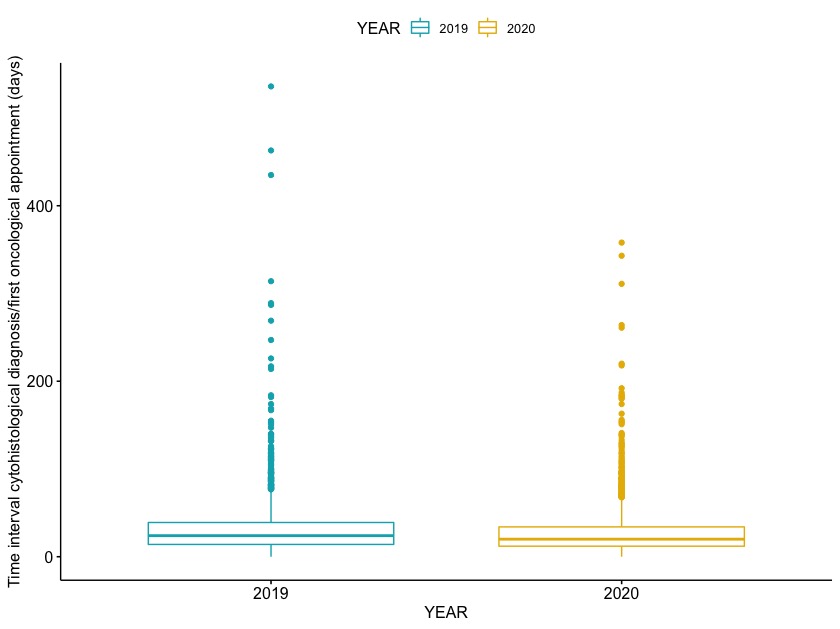

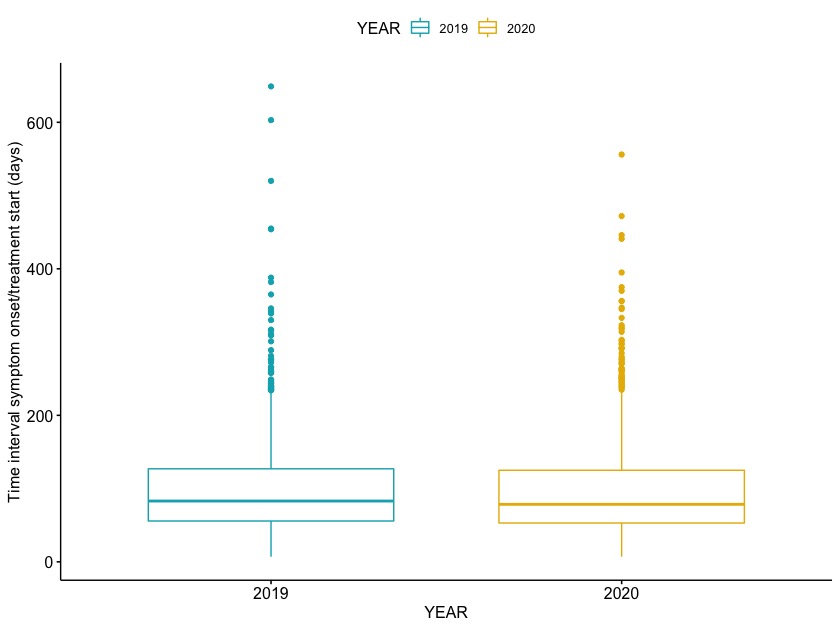

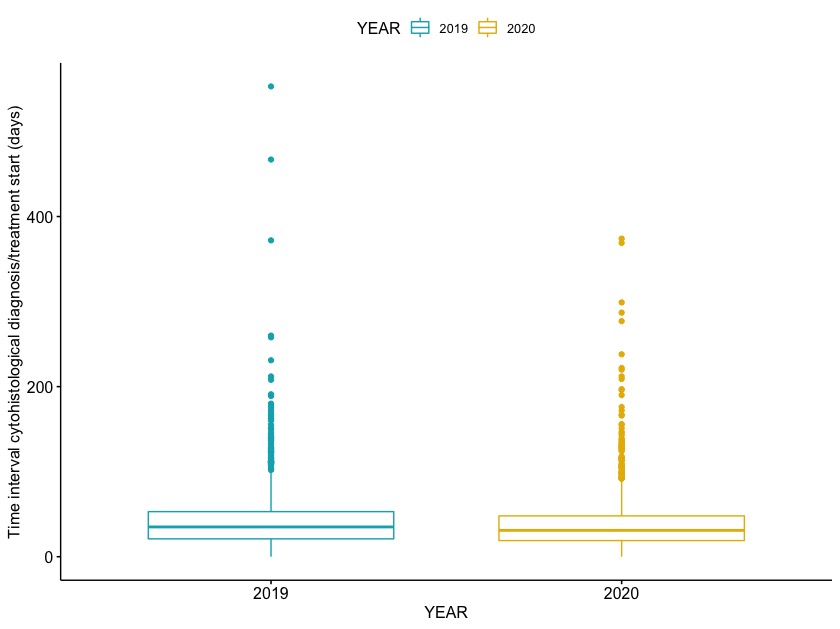

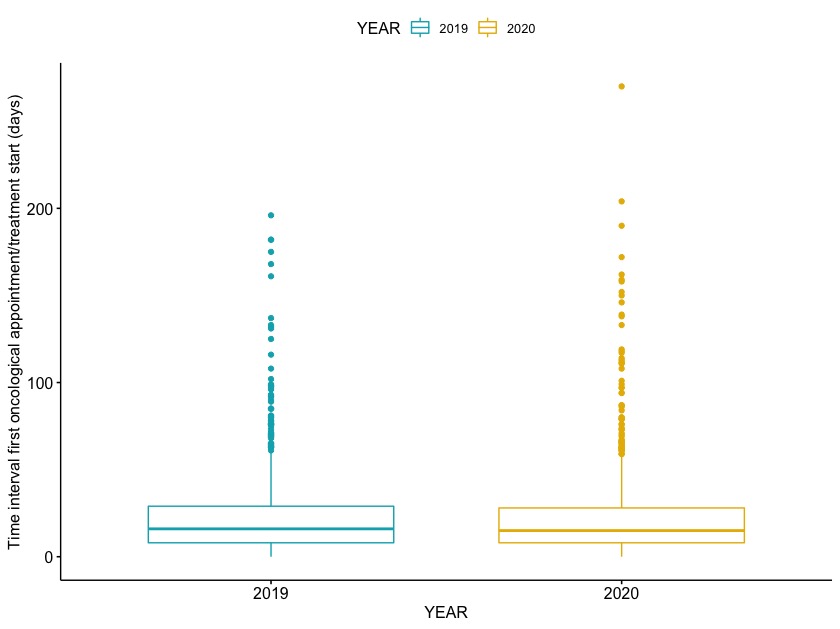

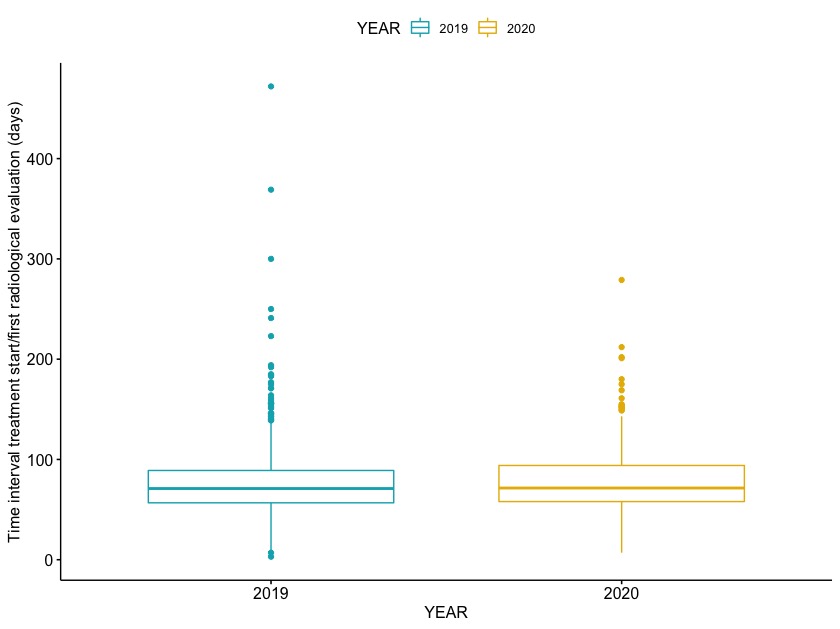
**
